# Face recognition algorithm using extended vector quantization histogram features

**DOI:** 10.1371/journal.pone.0190378

**Published:** 2018-01-02

**Authors:** Yan Yan, Feifei Lee, Xueqian Wu, Qiu Chen

**Affiliations:** 1 School of Optical-Electrical and Computer Engineering, University of Shanghai for Science and Technology, Shanghai, China; 2 Major of Electrical Engineering and Electronics, Graduate school, Kogakuin University, Tokyo, Japan; National University of Defense Technology, CHINA

## Abstract

In this paper, we propose a face recognition algorithm based on a combination of vector quantization (VQ) and Markov stationary features (MSF). The VQ algorithm has been shown to be an effective method for generating features; it extracts a codevector histogram as a facial feature representation for face recognition. Still, the VQ histogram features are unable to convey spatial structural information, which to some extent limits their usefulness in discrimination. To alleviate this limitation of VQ histograms, we utilize Markov stationary features (MSF) to extend the VQ histogram-based features so as to add spatial structural information. We demonstrate the effectiveness of our proposed algorithm by achieving recognition results superior to those of several state-of-the-art methods on publicly available face databases.

## Introduction

Face recognition, a typical biometric identification technology, is now recognized as an essential technology for establishing secure control. It has attracted much attention from researchers and engineers over the past decades owing to its wide range of applications in many fields, including information security, identity authentication, law enforcement, smart cards, access control systems and so forth. The entire face recognition procedure consists primarily of two operations: feature extraction and classifier design. These two steps have a substantial influence on the effectiveness and reliability of various recognition approaches. Regarding feature extraction, various face representation approaches have been discussed and studied, and all these approaches can be roughly divided into two categories: appearance-based methods and feature-based methods.

Appearance-based models use transformations and statistical methods to project samples from high-dimensional space into a much lower-dimensional feature subspace to extract the holistic features to represent the face. The Eigenfaces (PCA) [1] and Fisherfaces (LDA) [2,3] approaches are two of the most representative subspace techniques. The Eigenfaces approach, which is based on the Karhunen-Loeve transform, produces an expressive subspace for facial representation and recognition, while the Fisherfaces approach is a supervised subspace analysis technique that can search for the projection directions that are optimal for discrimination. More recently, some extensions of PCA and LDA based face recognition have been studied and applied, such as Two-dimensional PCA (2DPCA) [4] and Two-dimensional LDA (2DLDA) [5]. In contrast to Eigenfaces and Fisherfaces, which are based on one-dimensional image vectors, the newly proposed approaches are based on a two-dimensional (2D) image matrix that directly addresses the 2D face images without the need for image-to -vector transformation. Moreover, there are many other methods, such as Non-negative Matrix Factorization (NMF) [6], which—unlike PCA and LDA—is designed to capture the part-based structures inherent in the face images space. NMF is a method to obtain a data representation using non-negativity constraints. Locality Preserving Projections (LPP) [7] is an alternative approach to PCA. LPP is a linear subspace method that tries to optimally preserve the local neighbourhood information. Structure-Preserved Projections (SPP) [8] is an algorithm that takes the holistic context of a face into account and preserves the configural structure of each face image in subspace.

In contrast to the above subspace methods that directly consider whole-face images as the input patterns, feature-based methods are based on the relationships between local facial features such as the eyes, mouth, nose, and so on. Some commonly used feature-based methods exist. In [9], the Local Binary Patterns (LBP) method [9], feature histograms are extracted from each small region of facial images by considering each pixel in the image as well as the values of its neighbourhood pixels. The Histograms of Oriented Gradients (HOG) method [10, 11] has been shown to be an effective descriptor for object recognition in general, and it is particularly effective in face recognition tasks. In [12], Elastic Bunch Graph Matching (EBGM) [12] was proposed to recognize objects or object classes in an image based on a graph representation extracted from other images. Scale Invariant Feature Transform (SIFT) [13] is an algorithm used to detect and describe scale, translation and rotation-invariant local features in images. Other features can also be used for face recognition, such as Discrete Cosine Transform (DCT) [14], which has been used as a feature extraction step in various studies on face recognition, and Discriminative Common Vectors (DCV) [15], an approach proposed for face recognition that is based on a variation of Fisher’s Linear Discriminant Analysis for small sample sizes.

Aside from the above approaches, many other techniques exist to perform face recognition, such as Sparse Representation Classification (SRC) [16], Linear Regression Classification (LRC) [17], Vector Projection Classification (VPC) [18], Nearest Distance Classifiers (NDC) [19], Bayesian Classifier (BC) [20], Support Vector Machines (SVM) [15], Convolution Neural Network (CNN) [21] [22] and so on. Among these, the CNN has become one of the most popular techniques in recent years. There are numerous CNNs based methods, including AlexNet (one of the largest CNNs used in the ILSVRC-2010 competitions [23]) and CenterlossNet [21] (an optimized CNN architecture that utilizes a new supervision signal called centre loss to optimize CNNs. The discriminative deep features extracted from CenterlossNet have achieved excellent performances on several important face recognition benchmarks). Table 1 provides a summary of the acronyms and references of the algorithms mentioned above.

**Table 1:** Approaches mentioned in the Introduction.

Approach		Advantages	Limitations
Acronyms	Algorithms		
Ref.			
PCA	Eigenfaces	•Low computation time.	•Contains no class information on the input data.
[1]			•Fails to capture high-order statistics.
LDA	Fisherfaces	•Includes class-specific discriminatory information.	•Suffers from the small sample size problem.
[2, 3]			•Fails to capture high-order statistics.
2DPCA	Two-dimensional PCA	•Can directly extract the matrix features of 2D images.	•More coefficients than PCA for image representation.
		•Lower dimensionality than PCA.	
[4]		•More computationally efficient than PCA.	
2DLDA	Two-dimensional LDA	•Can directly extract the matrix features of 2D images.	•More coefficients than LDA for image representation.
[5]		•More computationally efficient and stable than LDA.	
NMF	Non-negative Matrix Factorization	•Can capture important local differences.	•Sensitivity to facial variations.
[6]			
LPP	Locality Preserving Projections	•Can find the intrinsic low-dimensional nonlinear manifold structure hidden in the observation space.	•Sensitivity to facial variations.
[7]			
SPP	Structure-Preserved Projections	•Can preserve the configural structure of facial image in subspace.	•Robust to variations such as head, pose, lighting condition, and facial expression.
[8]			
LBP	Local Binary Patterns	•Simple calculation.	•Sensitivity to noise.
		•Good for extracting the local texture features of a face image.	•Features contain no shape information.
[9]		•Invariant to rotation and grey-scale.	
HOG	Histograms of Oriented Gradients	•Invariant to illumination and 2D rotation.	•Non-robust to scale changes.
[10,11]			
EBGM	Elastic Bunch Graph Matching	•Can model a face as a 2-D elastic graph.	•Non-robust to changes in expression and illumination.
[12]			•High computation cost.
SIFT	Scale Invariant Feature Transform	•Robust to rotation and scale changes.	•High computation cost.
[13]			
DCT	Discrete Cosine Transform	•Data-independent.	•Complex calculation.
[14]		•Can be implemented using a fast algorithm.	
DCV	Discriminative Common Vectors	•Efficiency (real-time).	•Applications for under-sampled data are limited.
		•Numerical stability.	•Linear technique (Inadequate to describe the complexity of face image due to facial variations).
[15]		•Can handle the small sample size problem.	
SRC	Sparse Representation Classification	•Can correct corruptions possibly existing in testing data.	•Cannot handle cases in which the training data are corrupted.
[16]			• Computationally expensive.
LRC	Linear Regression Classification	•Simple architecture.	•Non-robust to severe illumination.
[17]		•Computationally efficient.	
VPC	Vector Projection Classification	•Simple architecture.	•Non-robust to severe illumination.
[18]		•Computationally efficient.	
NDC	Nearest Distance Classifier	•Computationally efficient.	•Lazy learning.
[19]			
BC	Bayesian Classifier	•Simple calculation.	•Need prior probabilities.
		•Few estimated parameters.	
[20]		•Insensitive to missing data.	
SVM	Support Vector Machines	•Efficient in classification with nonlinear data.	•Low efficiency in handling large-scale training samples.
[15]			•Low efficiency in solving multi classification problems.
CNN	Convolution Neural Network	•Robust to rotation, translation and scaling deformation of images.	•Require a large number of training samples.
[21] [22]			•Hardware requirements.

However, most of the face representation approaches mentioned above are subject to limitations, including computational issues, and can become quite complex. Although many of the appearance-based face recognition techniques work well in controlled environment, in many real-world applications, the number of available training samples is often limited. Consequently these techniques have difficulty handling substantial amounts of facial variations, such as changes in illumination, pose, accessories and expression, as well as performing sample analysis of new classes. For example, the conventional methods PCA and LDA rely on component analysis techniques. For classification purposes, LDA is generally considered superior to PCA when sufficient training samples per individual are available [2], however, when the number of available training samples per individual is far smaller than that we supposedly have, the experimental analysis in [24] indicates that PCA outperforms LDA. In particular, when one training sample per individual is available in the database, LDA cannot be readily applied because the within class scatter cannot be estimated. Moreover, most feature-based methods appear to be inadequate when nonrepresentative training samples are given. For example, the HOG descriptor [10] is non-robustness to scale changes, and the original LBP operator [9] suffers from sensitivity to noise and variance to rotation; thus, the applications of these methods in non-controlled environments are limited. Other face recognition techniques are quite complex and computationally expensive and are therefore unsuitable for processing the large numbers of training face images that are often required in practical applications. For example, Wright et al. [16] proposed a creative face recognition algorithm called SRC in which the occlusion matrix is an orthogonal matrix and the number of atoms required is very high. A large occlusion matrix can make the sparse coding process very computationally expensive and even prohibitive. Furthermore, most existing face representation approaches are hand-crafted and usually require strong prior knowledge for manual tuning. Therefore, in this paper, we focus on a featured-based algorithm and propose a simple yet effective approach for face recognition.

A reliable algorithm called the Vector Quantization (VQ) histogram method was developed in [25] to extract VQ histogram features of facial images for face recognition. Although the effectiveness of this method has been demonstrated by its excellent face recognition performances on the publicly available face database (the ORL database) [26], the inability of VQ histogram features to convey spatial structural information and take interactions between multiple different facial sub-regions into account greatly limits its discrimination capability—especially when applied to a larger face database. To overcome this limitation, in this paper, a novel recognition algorithm based on multiple image sub-regions (MSR-MSF-VQ) is proposed to address this problem. The key contributions of this paper are as follows:

1. Li et al. proposed a framework in [27] called MSF and utilized the framework to extend color histogram-based features with local location structure information. Based on this concept, we make full use of this framework and combine it with the VQ algorithm to incorporate spatial structural information into the VQ histogram. The obtained MSF extended VQ histogram features can be applied to face recognition and achieve satisfactory experimental results.
2. We propose the MSR-MSF-VQ algorithm based on the original MSF-VQ algorithm. The important characteristic of this extended algorithm is that it can consider the interactions of multiple different facial image sub-regions and thus preserve the significant location structure information and the spatial relationships of the facial sub-regions in the final feature information. The extended MSR-MSF-VQ features generated by our proposed algorithm can significantly improve face recognition performance.
3. During the process of face recognition, we can obtain satisfactory recognition results by simply transferring the comparison of two facial images to the comparison of two corresponding MSR-MSF-VQ features by using distance as a dissimilarity measure. We also make use of the advantages of the SVM classifier to further optimize the recognition performance.

The rest of the paper is organized as follows. First, we review the VQ and MSF approaches and then introduce the proposed MSR-MSF-VQ algorithm in detail. Next, we present the experimental results and analysis from tests on six well-known public face databases. Finally, conclusions are drawn.

## Material and methods

### Related algorithms

This section briefly reviews the VQ and MSF algorithms.

#### Vector quantization (VQ) histogram method

Feature extraction plays a crucial role in the face recognition process. VQ [25] is a widely used data compression model and an effective feature extraction algorithm that can extract VQ histogram features for face recognition. Here, we present a brief description of the VQ algorithm.

**Fig 1:**
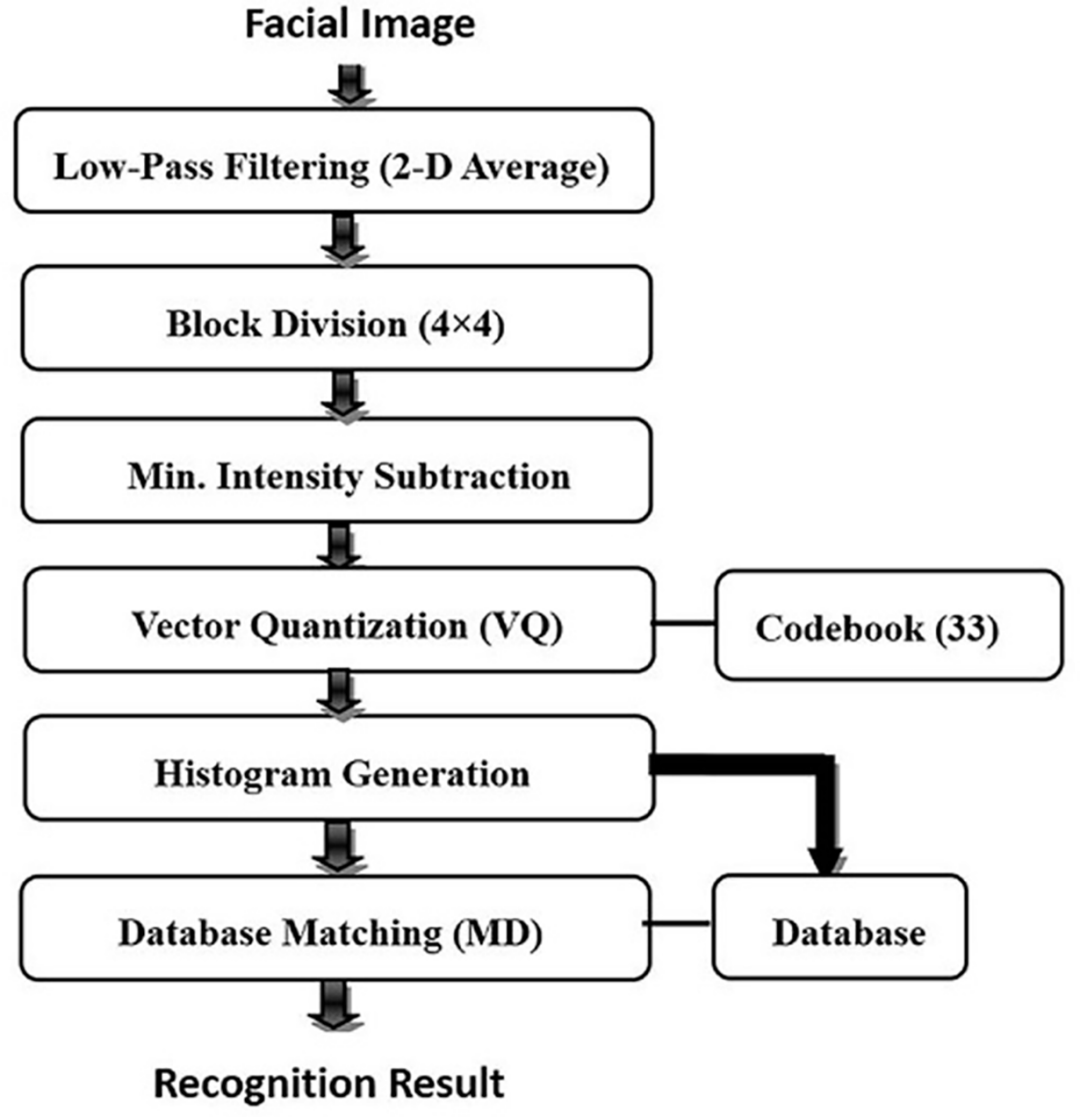
Face recognition process using the VQ algorithm.

As shown in Fig 1, we first use a simple 2-D moving average filter to preprocess the input image because low-pass filtering is essential for eliminating high-frequency noise and extracting the most effective low-frequency components for recognition. Next, a block division step is conducted. Specifically, this step divides the input image into 4×4 pixel blocks with a 15/16 overlap using a dividing-partition that slides through the pixels one by one. Then, the minimum intensity in each 4×4-pixel block is found and subtracted from each pixel in the block. This effectively compensates for all brightness variations while preserving the intensity variations in the block for further feature extraction processing. The blocks with varying intensity are taken as input vectors, each of which is matched with the codevectors in a codebook containing 33 codevectors. By computing the Manhattan distances between the input vectors and the codevectors, we match the most similar codevector to the input vector by finding the minimum distance. After performing VQ for all the blocks in the image, a VQ histogram is generated by counting the matched frequencies for each codevector. In the registration procedure, we apply the VQ algorithm to all the facial images in a set of images and utilize their VQ histogram features as personal identification information to establish a face database. Subsequently, in the recognition procedure, the VQ histogram created from an unknown given facial image is compared with the registered individual VQ histograms, and the best match is output as the recognition result. The corresponding experimental results illustrated in [25] have demonstrated the effectiveness of the VQ histogram algorithm.

#### Markov stationary features (MSF)

As described in [27], we know that MSF can essentially handle three-level histogram-distinguishable problems; thus, they can alleviate the limitations of histograms. We can utilize this framework to extend the histogram-based features with spatial structural information from the facial image. Therefore, in the following, we provide an overview of Markov stationary features.

Let *p*_*k*_ denote a pixel in image I. *C* = (*c*_*ij*_)_*K*×*K*_ represents the spatial co-occurrence matrix, each element of which takes the following form:

$$c_{ij}=\#\left(p_{1}=c_{i},p_{2}=c_{j}|\left|p_{1}-p_{2}\right|=d\right)/2$$ (1)

where d denotes the distance between the pixels *p*_1_ and *p*_2_, and *c*_*ij*_ counts the number of spatial co-occurrences for bins *c*_*i*_ and *c*_*j*_.

After obtaining the co-occurrence matrix, the corresponding transition matrix *P* = (*p*_*ij*_)_*K*×*K*_ derived from the spatial co-occurrence matrix *C* = (*c*_*ij*_)_*K*×*K*_ can be easily computed using Formula (2), in which *p*_*ij*_ denotes the probability of changing from state *c*_*i*_ to *c*_*j*_.

$$p_{ij}=\frac{c_{ij}}{\sum_{j=1}^{K}c_{ij}}$$ (2)

Suppose the state distribution after *n* steps is *π*(*n*) and the initial distribution is *π*(0). The stationary distribution is an invariant measure of a Markov chain, which can be accumulated by:

$$A_{n}=\frac{1}{n+1}\left(I+P+\cdots +P^{n}\right)$$ (3)

$$\pi \approx \frac{1}{K}\sum_{i=1}^{K}{\bar{a}}_{i},\;where\;A_{n}={\left[\vec{a_{1}},\cdots ,\vec{a_{K}}\right]}^{T}.$$ (4)

Here, *π* is the stationary distribution that satisfies *π* = *πP*.

Finally, the complete MSF feature which includes the combination of the initial distribution defined by Formula (5) and the stationary distribution can be obtained by Formula (6).

$$\pi (0)=\frac{c_{ii}}{\sum_{i=1}^{k}c_{ii}}$$ (5)

$$\vec{MSF}={\left[\pi (0),\pi \right]}^{T}$$ (6)

### The proposed MSR-MSF-VQ algorithm

Although VQ was shown to be effective in [25], some room exists for improvement because the original version does not capture any spatial structural information. Considering this lack, [28] developed the MSF-VQ algorithm by combining MSF with the original VQ algorithm to encode spatial structural information into VQ histogram-based features to improve the accuracy of a facial recognition system. The combined MSF-VQ features are key in implementing the MSF-VQ algorithm for facial recognition and can be achieved by following these five steps.

Step 1: Conduct a simple a low-pass filtering to preprocess the input facial image to reduce high-frequency noise and extract the most effective low-frequency component for face recognition.

Step 2: Quantize the facial sub-region into 33 levels utilizing the VQ method.

Step 3: Construct a spatial co-occurrence matrix based on a defined distance *d*.

Step 4: Calculate the Markov transition matrix, which is derived from the spatial co-occurrence matrix.

Step 5: Normalize the self-transition as the initial distribution and combine it with the stationary distribution to obtain the complete MSF-VQ feature.

After obtaining the MSF-VQ features, the next step is the face recognition procedure, which is similar to the original VQ algorithm. We can obtain the final recognition results by transferring the comparison of two facial images to the comparison of two corresponding MSF-VQ features. Although the recognition results using MSF-VQ algorithm on the ORL database were reported in our earlier work [28] are better than that of the original VQ algorithm, thus demonstrating the effectiveness of the MSF-VQ algorithm, there are still some limitations in the MSF-VQ features, because the MSF-VQ features generated from the full facial image contain no location information concerning the facial sub-regions. This lack can degrade the face recognition performance—especially when the MSF-VQ algorithm is applied to a large face database such as FERET, which is larger than the ORL database. Considering this aspect, we felt sure that further research on the original MSF-VQ algorithm could yield more powerful discrimination capability for facial recognition. Therefore, in this paper, an extended version of the MSF-VQ algorithm based on several image sub-regions, called MSR-MSF-VQ, is proposed to address this problem. In contrast to the original MSF-VQ algorithm, this new proposed MSR-MSF-VQ algorithm not only retains the advantages of the MSF-VQ algorithm but also integrates the location information and spatial relationships of facial sub-regions into the MSF-VQ features to obtain a better facial feature representation for face recognition.

**Fig 2:**
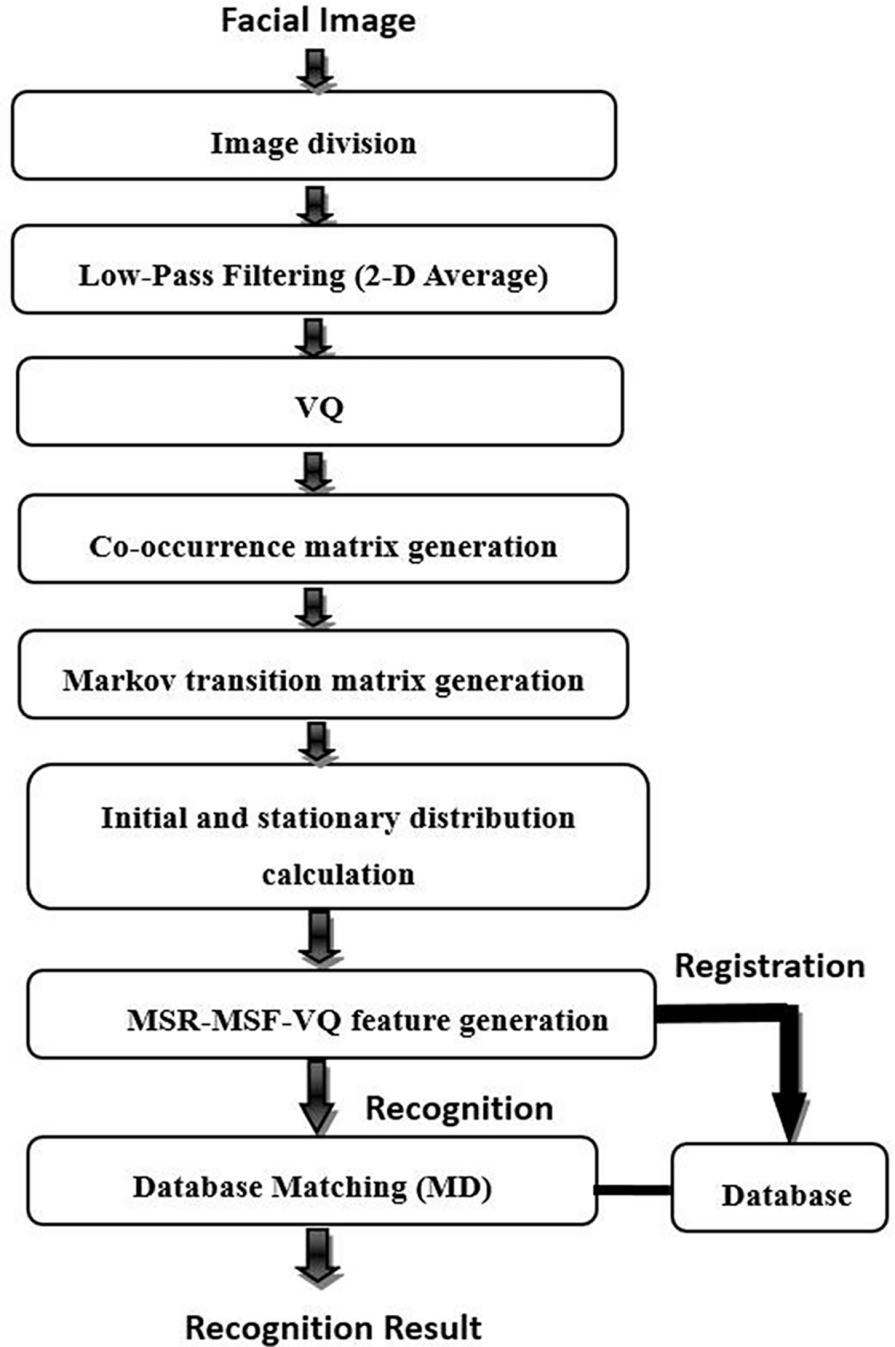
Face recognition process using the MSR-MSF-VQ algorithm.

**Fig 3:**
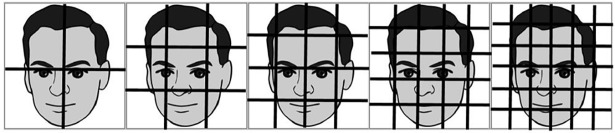
Face image partition strategies based on several equal-sized sub-regions.

Fig 2 shows the face recognition process using our proposed MSR-MSF-VQ algorithm. More specifically, in the implementation process of our proposed algorithm, after normalization, the facial image is first divided into several sub-regions (Fig 3 shows examples of segmenting a face image into several equal-sized sub-regions in a non-overlapping way). Then, the individual MSF-VQ features of each sub-region can be extracted independently through the series of steps and formulas mentioned above. After obtaining the MSF-VQ features, the comparison of each same sub-region of two facial images can be transferred to the comparison of two corresponding MSF-VQ features using a dissimilarity measure method; here, we choose Manhattan distance as the matching measure following [28], which can be computed using Formula (7). The symbols $\vec{h_{r}(A)}$ and $\vec{h_{r}(B)}$(r represents each sub-region) stand for the MSF-VQ features belonging to each sub-region of facial images derived from the gallery and probe sets. By concatenating the recognition results based on different facial sub-regions using weighted averaging, the newly generated MSR-MSF-VQ feature is finally formed and can be utilized as a substitute for the original MSF-VQ features for face recognition. The formula used during the face recognition process is given in Formula (8), in which the symbol *w*_*r*_ represents the corresponding weighting factor of the MSF-VQ feature for each facial sub-region.

$$D(\vec{h_{r}(A)},\vec{h_{r}(B)})=\sum_{i=1}^{66}|\vec{h_{r(i)}(A)},\vec{h_{r(i)}(B)}|$$ (7)

$$D(\vec{h(A)},\vec{h(B)})=\frac{w_{r}\times D(\vec{h_{r}(A)},\vec{h_{r}(B)})}{\sum w_{r}}$$ (8)

The related experimental results and comparative analyses using MSR-MSF-VQ algorithm for face recognition are presented in the next section.

## Results and discussion

In this section, to evaluate the feasibility and performance of our proposed algorithm, we carried out experiments on six standard public face databases: ORL [26] [29], FERET [30] [31], AR [32], Yale [33], Yale- B [34, 35] and CAS-PEAL-R1 [36, 37]. These databases all contain face images with pose, expression, illumination and occlusion variations. The first database was used to choose appropriate parameters for the MSF-VQ algorithm. Then, the FERET, AR, Yale, Yale—B and CAS-PEAL-R1 databases were used to compare and evaluate our new proposed MSR-MSF-VQ algorithm with previous face recognition approaches. The details of the corresponding experiments and the results will be given in the following subsections. Our proposed algorithm was programmed using ANSI C and executed on a PC with an Intel(R) Xeon(R) E5-2620 CPU running @ 2.1 GHz with 32 GB RAM and a Linux (Fedora distribution) operating system.

### Determination of the parameters

To apply the MSF-VQ algorithm, several parameters such as the direction of the occurrence matrix, *d* (the distance used in the co-occurrence matrix), and *n* (the number of transfer times) must be determined in advance. This subsection contains an analysis of how to set these parameters. We conducted experiments on the ORL face database to investigate the sensitivities of these three parameters. The ORL database [25] [29] contains 400 facial images of 40 different people; there are 10 images of each person in different poses and with different expressions. All the images in this database are greyscale and have a resolution of 92 × 112 pixels (The facial images in the ORL face database are copyrighted, which limits the publication of these facial images in PLOS ONE for commercial use. Consequently, in this paper, we have removed the image samples from the ORL face database). In our experiments, we used five images from each individual for training and the remaining five images for testing by using the rotation method. Thus, in total, there are 252 ($C_{10}^{5}$) training-testing combinations. The final recognition rates are obtained by taking the mean of the 252 recognition results.

From the previous work described in [28], we know that we can utilize different directions of the Markov stationary features to eliminate the inherent ambiguity associated with MSF caused by the symmetric property of the co-occurrence matrix. Table 2 presents the corresponding experimental results. The symbols such as “MSF-VQ (0)”, “MSF-VQ (90)”, “MSF-VQ (45)”, and “MSF-VQ (135)” stand for the MSF-VQ algorithm based on the horizontal case, vertical case and two diagonal cases, respectively, meanwhile, the symbols related to “MSF-VQ (mix)” and MSF-VQ (ave) separately represent the cases that use the co-occurrence matrix based on the four directions and those that use different MSF-VQ features based on the four directions with weighted average. From Table 2, we can see that the recognition accuracy of 96.15% achieved by the MSF-VQ (ave) algorithm constitutes an improvement of the recognition rate compared with the original VQ algorithm, which indicates the effectiveness of the MSF-VQ algorithm when considering the influences of different directions.

**Table 2:** Face recognition accuracy based on seven cases.

Approach	Recognition rate (%)
VQ	95.600
MSF-VQ(0)	95.435
MSF-VQ(45)	95.442
MSF-VQ(90)	96.278
MSF-VQ(135)	96.254
MSF-VQ(mix)	95.722
MSF-VQ(ave)	96.153

**Fig 4:**
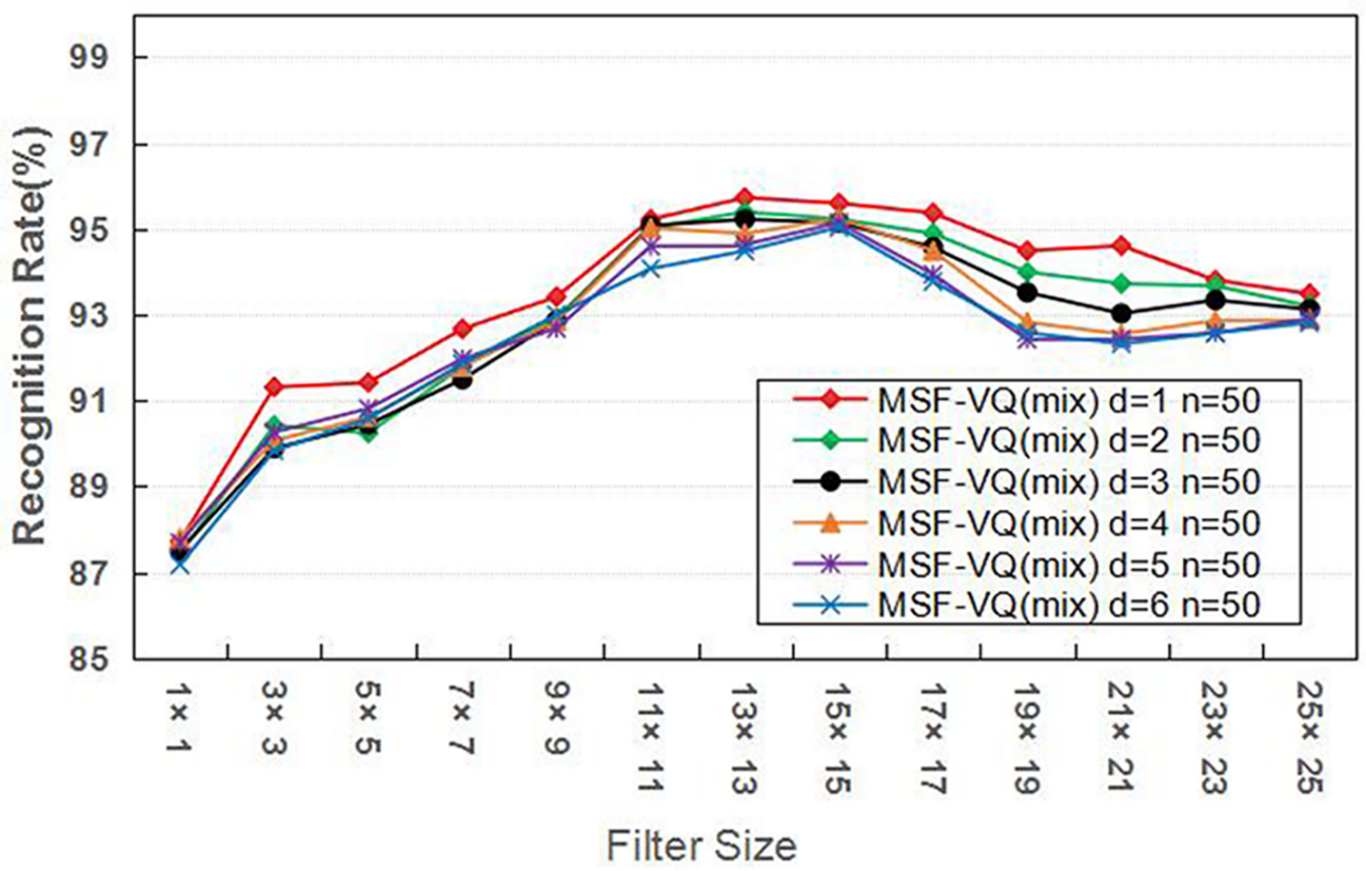
The average recognition rate using different values of d.

**Fig 5:**
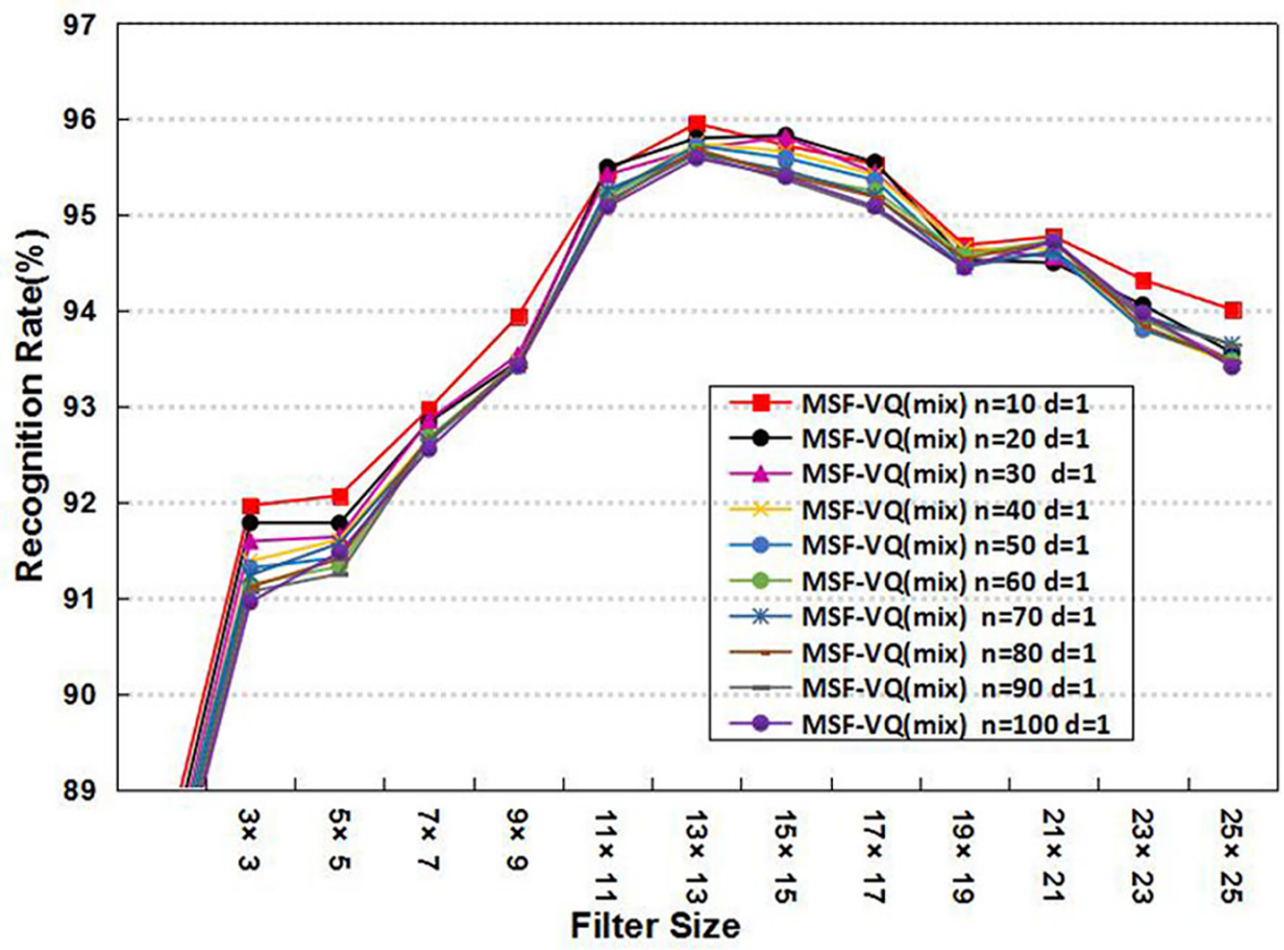
The average recognition rate using different values of n.

Furthermore, considering the essential role of the parameters related to *d* and *n* in our MSF-VQ algorithm, we performed two experiments using the MSF-VQ (mix) algorithm on the ORL database to select optimal values for the parameters *d* and *n*. In the first experiment, we fixed *d* to 1 and varied *n* from 10 to 100. In the second experiment, we fixed *n* to 50 and varied *d* from 1 to 6. We calculated the recognition rate for all variations. The corresponding average recognition results are shown as a function of filter size in Fig 4 and Fig 5, respectively. Both reflect the same trend: the average recognition rate first increases as the filter size increases, and then becomes saturated or gradually decreases. In more detail, the experimental results in Fig 4 show that the optimal recognition performance is obtained when *d* is fixed to 1, while Fig 5 shows that the impact of parameter *n* on the face recognition rate is not obvious, especially at filter sizes of 11×11 or 13×13. This result confirms that it is feasible and suitable to choose *n* = 50 (the same value as was used in [27]) and *d* = 1 in our algorithm.

### Experiments on the FERET face database

The experimental results on ORL face database demonstrated the effectiveness of the algorithm combining MSF and VQ. To more convincingly demonstrate the effectiveness of the MSF-VQ algorithm, we applied it to a larger face database called FERET [30] [31] [38] and evaluated the resulting recognition accuracy. The FERET database contains 14,051 greyscale facial images that include variations in scale, illumination, pose and facial expression. The resolution of each image is 256×384. In our next set of experiments, we tested our algorithm using the FB section of the FERET face database. This task has been widely used to evaluate facial recognition accuracy. In more detail, the database contains 1,196 frontal images in the fa set and 1,195 frontal images in the fb set. Each set contains only one image per person. The fa set consists of different facial expressions from fb; we selected fa as the gallery set and fb as the probe set. All the input facial images are normalized and resized to 146×200 pixels utilizing the two eye coordinates supplied by the FERET face database (The facial images in the FERET face database are copyrighted, which limits the publication of these facial images in PLOS ONE for commercial use. Consequently, in this paper, we have removed the image samples from the FERET face database).

Our previous experimental results reported in [39] validated the effectiveness of the MSF-VQ algorithm on the FERET face database. However, compared with some state-of-the-art algorithms, the recognition performance of the MSF-VQ algorithm was still far from ideal because the MSF-VQ features of the whole-face image contain no location information or any spatial relationships of the facial sub-regions. Therefore, we planned to apply the new proposed MSR-MSF-VQ algorithm to our face recognition task to address this problem. We expected to obtain an improved face recognition performance.

**Fig 6:**
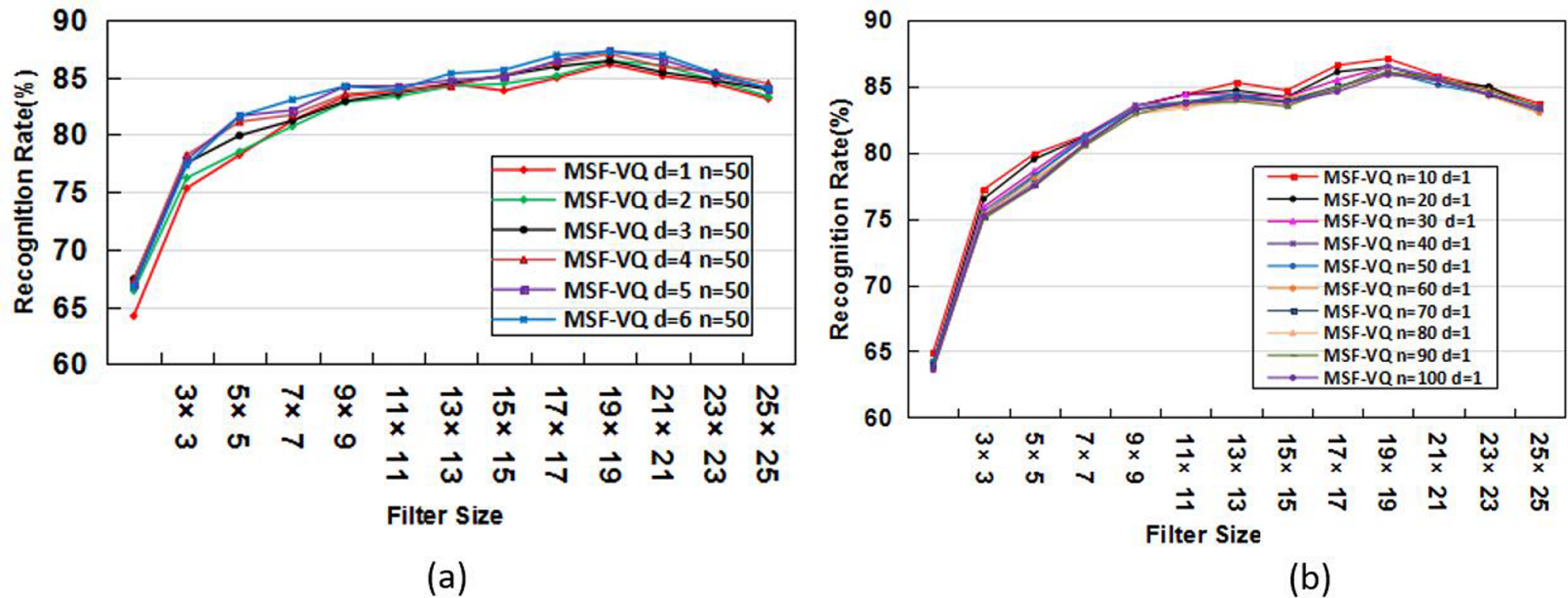
(a) Face recognition rate achieved by varying d and fixing n = 50 on the FERET database. (b) Face recognition rate achieved by varying n and fixing d = 1 on the FERET database.

Before validating the effectiveness of our new proposed MSR-MSF-VQ algorithm, we investigated the impacts of factors such as image size (F1), similarity measures (F2), and directions of the occurrence matrix (F3).These parameters play essential roles in our algorithm; consequently, to obtaining the optimal parameter values would facilitate our future work. Moreover, we also conducted experiments to further investigate the sensitivity of the parameters (*d* and *n*) by testing using a larger database than the ORL database. Our recognition results on the FERET database are shown in Fig 6. These results reveal similar experimental phenomena with the results on the ORL database. Therefore, we can conclude that these two parameters (*d* and *n*) are not sensitive to the training database. Therefore, in the next set of experiments based on other face databases, the parameters *d* and *n* are fixed and set as they were in this work. Next, we vary the factors F1, F2 and F3. The experimental results are listed in Table 3, from which we can observe that based on these factors, the MSF-VQ algorithm shows only slight differences in the face recognition accuracy. Therefore, we still adopt the Manhattan distance as the similarity measure, select the facial identification feature using four directions, and use the original image size (146×200 pixels) for our face recognition task.

**Table 3:** Influence of different factors on the face recognition rate using the MSF-VQ algorithm.

Image size											
Zoom Factor	0.5	0.6	0.7	0.8	0.9	1	1.1	1.2	1.3	1.4	1.5
Rank-one recognition rate (%)	82.8	83.9	84.4	84.8	85.2	86.1	86.5	86.6	86.9	86.0	86.0
Similarity measures											
Measure Method	Euclidean				Manhattan			Formula in [27]			
Rank-one recognition rate (%)	84.6				86.1			84.5			
Directions of the occurrence matrix											
Direction	0	45		90		135		mix		ave	
Rank-one recognition rate (%)	85.2	85.8		86.7		85.5		86.1		85.9	

Next, we conducted experiments based on different division strategies to investigate the effectiveness of our proposed MSR-MSF-VQ algorithm. In practice, when implementing our MSR-MSF-VQ algorithm, the resized square face images are first partitioned into several equal image sub-regions; then we apply the MSF-VQ algorithm to each equal-sized image sub-region to obtain the individual recognition results. These results are concatenated by weighted averaging in the face recognition procedure to obtain the final combined MSR-MSF-VQ features for face recognition. Table 4 shows the experimental results from applying different image division strategies. The 1st column lists the division strategies; the 2nd column shows the size of face image; and the 3rd column presents the maximum recognition rate using the MSR-MSF-VQ algorithm based on the different division strategies. From Table 4, we can conclude that the recognition rate increases as the division strategy changes from 1×1 sub-regions to 5×5 sub-regions, however, this increasing trend is not maintained when more precise division strategies are used for feature extraction. After segmenting the face images into 5×5 sub-regions with a size of 41×41 pixels and achieve the maximum recognition rate of 98.2%, the face recognition accuracy decreases. This occurs because more precise division strategies introduce additional noise, which is not beneficial for the recognition performance. Only by combining different MSF-VQ features based on several image sub-regions using an appropriate division strategy will the recognition rate be improved. In contrast to the previously introduced MSF-VQ algorithm, applying the MSR-MSF-VQ algorithm results in a considerable improvement in recognition accuracy. The largest contribution of our method is that it considers the interactions of multiple different facial image sub-regions. This approach more accurately describes the content of the facial images and preserves more significant personal identification information during feature extraction, which leads to the final excellent face recognition performance.

**Table 4:** Recognition results using different segmentation strategies on the FERET database (results obtained with our proposed algorithm are in bold).

Division strategies	Image size	Rank-one Recognition rate (%)
1×1	204×204	86.3
2×2	204×204	95.3
3×3	204×204	97.3
4×4	204×204	97.5
**5×5**	**205×205**	**98.2**
6×6	204×204	98.1
7×7	203×203	97.8

To clearly present the effectiveness of our proposed MSR-MSF-VQ algorithm, we compared it with various state-of-art approaches using the same FB task of the FERET database. The compared approaches include PCA [1] (considering Euclidean and Mahalanobis Cosine distances), LDA [2], the Bayesian algorithm with variants MAP and ML [20], Gabor-EBGM [12], HOG [10], HOG–EBGM [11], LBP [9] and SIFT [13]. The results listed in Table 5 show that the MSR-MSF-VQ algorithm achieves state-of-the-art accuracy, which validates the effectiveness of our proposed algorithm.

**Table 5:** Performance comparison on the FERET face database (results obtained with our proposed algorithm are in bold).

Approach	Rank-one Recognition rate (%)
LDA	72.1
Bayesian MAP	81.7
Bayesian ML	81.7
PCA Mahalanobis Cosine	85.3
Gabor-EBGM	87.3
HOG	90.0
LBP	93.0
PCA Euclidean	94.3
HOG-EBGM	95.5
SIFT	95.9
**MSR-MSF-VQ**	**98.2**

Furthermore, given that deep learning—in particular, the convolutional neural network (CNN) that is widely used in computer vision community—has achieved promising results in face recognition recently, we compared our proposed algorithm with some recent works based on CNNs to further validate the effectiveness of our proposed algorithm. We conducted extensive experiments based on CNNs, still using the FB task in the FERET database for face recognition.

Because we lacked large-scale datasets to train our own deep learning model, we used pre-trained models and CNN architectures and then fine-tuned the parameters on our own dataset. We mainly chose two CNN architectures (AlexNet and CenterlossNet) for our experiments. AlexNet [22] is one of most representative convolutional neural networks. It can classify the 1.2 million high-resolution images in the ImageNet LSVRC-2010 contest into 1000 different classes. The pre-trained AlexNet model used here was supplied by Krizhevsky et al. [22], and was trained on a subset of ImageNet with approximately 1000 images in each of 1000 categories. In total, there are approximately 1.2 million training images, 50,000 validation images, and 150000 testing images. CenterlossNet (a newly proposed CNN) is an optimized CNN jointly supervised by the softmax loss and the center loss. As demonstrated in [21], compared with most recent works based on Deep Learning such as FaceNet and DeepFace, CenterlossNet can achieve excellent recognition performance with much less training data and a simpler network architecture. The pre-trained model supplied by Wen et al. [21] was trained on web-collected training data including the CASIA-WebFace, CACD2000, and Celebrity+ image databases. In our experiments, we used fine-tuned models (8000 iterations) of these two models (AlexNet and CenterlossNet) to extract deeply learned features for face recognition and compare our proposed algorithm with these models to further validate the effectiveness of our proposed algorithm.

To compare the performance of our proposed algorithm with the methods based on CNNs, for the face recognition process, we not only used the previously introduced Manhattan distance as the similarity measure to obtain the recognition results but also adopted the Support Vector Machine (SVM) [15] to optimize the face recognition performance. SVM is a widely used classifier that employs a supervised pattern recognition scheme method with two significant features: (1) SVM achieves an optimal linear classifier (optimal hyperplane) in the feature space whose training process involves a linear classifier with minimum machine complexity, thereby keeping the expected generalization errors low. (2) SVM makes efficient use of extremely high dimensional feature spaces using kernel functions. In our experiments, we applied the LIBLINEAR model supplied by [40] to obtain our recognition results (the kernel function was the radial basis function (RBF), and the C (penalty factor) equalled 0 ~ 10). In addition, the MSR-MSF-VQ face image features used in our experiments were represented by concatenating the MSR-MSF-VQ features extracted from different sub-regions (we adopted a division strategy of 5 × 5 sub-regions and the dimension of the MSR-MSF-VQ features was 1650). These features were utilized to obtain the final face recognition results based on the distance measure and the SVM classifier.

Table 6 shows the corresponding experimental results. The symbol “MSR-MSF-VQ+SVM” represents the MSR-MSF-VQ algorithm plus the SVM classifier). As Table 6 shows, compared with the original distance measure, the SVM classifier improves the recognition results. Our proposed MSR-MSF-VQ algorithm's results are higher than those of CenterlossNet and AlexNet, which indicates the effectiveness of our proposed algorithm.

**Table 6:** Recognition rates of different approaches on the FERET database.

Methods	Feature dimension	Recognition Accuracy (%)
AlexNet+Manhattan	4096	89.5
AlexNet+SVM	4096	93.97
CenterlossNet+Manhattan	512	97.0
CenterlossNet+SVM	512	99.0
MSR-MSF-VQ+Manhattan	1650	98.2
MSR-MSF-VQ+SVM	1650	99.16

Finally, we compared the average execution time of our proposed algorithm with the times of the different approaches mentioned in Table 7. Note that the processing time for a single image using our proposed algorithm on the FERET database is 1,883 ms, which includes 50 ms for preprocessing (including filtering and image preprocessing), 69 ms for VQ processing, 381 ms for feature extraction, and 1,383 ms for face recognition (the gallery set contains a total of 1196 face images). As Table 7 shows, the MSR-MSF-VQ algorithm is more efficient than the other methods in terms of the feature extraction time and the total execution time for face recognition.

**Table 7:** Recognition times of different approaches.

Method	Feature extraction time (ms)	Total time (ms)
MSF-VQ	381	1883
AlexNet	1360	47795
CenterlossNet	1917	9587

### Experiments on the AR face database

The AR face database [32, 41] contains more than 4,000 images of 126 different subjects (70 male and 56 female) (The facial images in the AR face database are copyrighted, which limits the publication of these facial images in PLOS ONE for commercial use. Consequently, in this paper, we have removed the image samples from the AR face database). We can see that the images in the AR face database have various facial expressions (neutral, smiling, angry), the lighting varies (e.g., brightly lit), and some of the images are partially occluded by sunglasses and scarves. Consequently, the AR database is more challenging. In these experiments, we take on the challenge of face recognition to test our proposed algorithm robustness to partial occlusion.

For our experiments, we selected 1,300 images of 100 individuals (50 males and 50 females)—13 different images for each subject. All the images are greyscale and cropped to 90×120 pixels based on two eye centre coordinates. To verify the effectiveness of our algorithm, we designed two test sets (the first comprised 300 scarf-occluded images and the second comprised 300 sunglasses-occluded images) and utilized the remaining seven images for each subject to create the training set. Table 8 contains a detailed comparison of the original MSF-VQ with a variety of state-of-the-art approaches. From Table 8, we can see that the recognition results of the original MSF-VQ lag those of the algorithms SRC [16], LRC [17] and VPC [18]; however, for the scarf-occluded images, the results of MSF-VQ exceed those of the other methods except for Fisherfaces and SRC.

**Table 8:** The recognition rates of different approaches on the AR database.

Approach	Sunglasses (%)	Scarf (%)
DCV	13.33	10
Fisherfaces	27.33	23.67
DCT	29.67	5.33
Eigenfaces	42.33	7.33
**MSF-VQ**	**54**	**13.7**
SRC	55.67	27.67
LRC	59	9.33
VPC	62.67	6.67

Next, we conducted experiments to demonstrate the effectiveness of the proposed MSR-MSF-VQ algorithm under partial occlusion conditions using the same training and test sets. Table 9 reports the comparison results of MSR-MSF-VQ algorithm with LVPC [18] and MLRC [17] (LVPC and MLRC are extended versions of VPC and LRC, respectively, that divide the images into four sub-regions). The symbols “MSR-MSF-VQ-4”, “MSR-MSF-VQ-16”, and “MSR-MSF-VQ-25” represent the MSR-MSF-VQ algorithm using three differently sized partitioning strategies. On the sunglasses-occluded images, the MSR-MSF-VQ-4 algorithm achieves the highest recognition (89%), greatly outperforming the other methods. However, on the scarf-occluded images, it falls behind the other algorithms. However, by applying more precise division strategies, the recognition rate increases significantly, exceeding all the other methods both cases (sunglasses and scarves). This is particularly evident when we employ the SVM classifier; the recognition rate rises to 100% for both types of occluded images.

**Table 9:** The recognition rates of different approaches and different partitioning strategies.

Approach	Sunglasses (%)	Scarf (%)
MLRC	67.33	95.33
LVPC	70.00	83.33
MSR-MSF-VQ-4	89.00	29.70
MSR-MSF-VQ-16	100	94.30
MSR-MSF-VQ-25	99.7	98.0
**MSR-MSF-VQ-SVM**	**100**	**100**

These experimental results clearly reflect that using whole-face MSF-VQ features is not a good strategy for face recognition and leads to uncompetitive recognition performances. However, by deploying an appropriate division strategy and using the combined MSF-VQ features for face recognition, the recognition performance improves. The satisfactory performance achieved on the AR face database confirms that our MSR-MSF-VQ algorithm is robust to partial occlusions.

### Experiments on the Yale face database

To further explore the performance of the MSR-MSF-VQ algorithm under facial expression and illumination variations, we applied it to the Yale database [33], which consists of 165 frontal greyscale images of 15 subjects, with 11 different images for each person. All the images are normalized to 100×100 pixels. More specifically, we used this database to evaluate the recognition accuracy of our proposed MSR-MSF-VQ algorithm under conditions where facial expressions (normal, happy, sad, sleepy, surprised and winking), occlusions (with and without glasses) and illumination (centre, left and right lighting) vary. (The facial images in the Yale face database are copyrighted, which limits the publication of these facial images in PLOS ONE for commercial use. Consequently, in this paper, we have removed the image samples from the Yale face database).

For our first experiment, we randomly chose six images as gallery sets and the remaining five images as probe sets for each person, similar to [8] (hence, 90 images were used for training and 75 images for testing). This random selection operation was repeated 10 times. Table 10 shows the average recognition results of the MSR-MSF-VQ algorithm and several subspace analysis methods. The compared algorithms are listed in Table 11. Among these, PCA, NMF, LPP, and S-LPP are holistic feature extraction models, while the others are all local matching algorithms. The experimental results show that all the local matching methods achieved better recognition performances than do the holistic methods. This occurred because the holistic methods can only extract global features for face recognition, which causes their performances to be deeply affected by pose, lighting condition, and facial expression variations in the facial images. The results also show that our proposed MSR-MSF-VQ algorithm significantly outperformed all the local matching methods, because the MSR-MSF-VQ algorithm considers the interactions between different sub-regions, which causes the facial recognition features to include both the location information and the spatial relationships of facial sub-regions. Therefore, the MSR-MSF-VQ algorithm achieves better recognition performance than the other methods.

**Table 10:** Performance comparison on the Yale face database (results of our proposed algorithm are in bold).

Approach	Rank-one Recognition rate (%)
LPP	75.00
S-LPP	75.73
NMF	77.27
SubXPCA	77.80
PCA	78.00
ModPCA	83.47
SpPCA	83.87
Aw-SpPCA	84.93
SpSLPP	86.13
SpNMF	88.40
SPP	93.33
**MSR-MSF-VQ**	**98.40**

**Table 11:** Algorithms compared in our experiments on the Yale database.

No.	Algorithms	Acronyms
1	Principle Component Analysis	PCA [1]
2	Modular Principle Component Analysis	ModPCA [42]
3	Sub-pattern Principle Component Analysis	SpPCA [43]
4	Adaptively Weighted Sub-pattern Principle Component Analysis	Aw-SpPCA [44]
5	Cross-sub-pattern correlation based Principle Component Analysis	SubXPCA [45]
6	Non-negative Matrix Factorization	NMF [6]
7	Sub-pattern Non-negative Matrix Factorization	SpNMF [46]
8	Sub-pattern based Spatially Smooth Locality Preserving Projections	SpSLpp [47,48]
9	Locality Preserving Projections	LPP [7]
10	Spatially Smooth Locality Preserving Projections	S-LPP [47]
11	structure-preserved projections	SPP [8]

**Fig 7:**
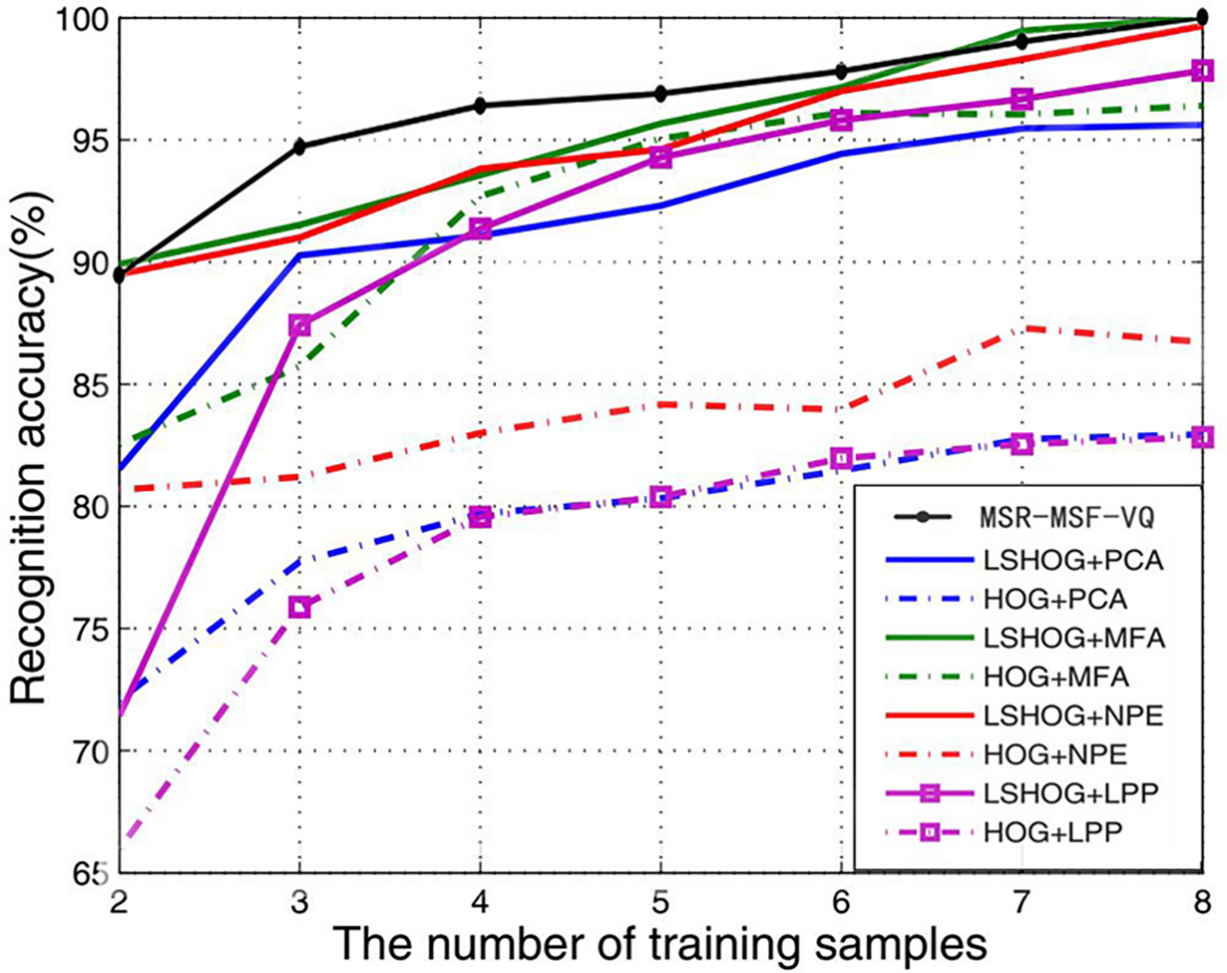
Mean recognition accuracy comparison on the Yale face database.

In our second set of experiments with the Yale database, we varied the number of the training samples. More specifically, we selected m (m = 2, 3…, 8) images of each person from the Yale face database as the training set and employed the remaining (11-m) images for testing. For each m we repeated our face recognition experiments 50 times using the MSR-MSF-VQ algorithm, and calculated the mean of the 50 results. The corresponding experimental graph of our proposed algorithm compared with LSHOG (locality sensitive histograms of oriented gradients) [49] and HOG [10] plus different dimension reduction algorithms including PCA [1], MFA [50], NPE [51] and LPP [7] using the same Yale face database are plotted in Fig 7. The Y-axis denotes the recognition accuracy and the X-axis shows the number of training samples. The results show that the recognition performance of LSHOG is superior to that of HOG regardless of what type of dimension reduction algorithm is used. Furthermore, Fig 7 also shows that the MSR-MSF-VQ algorithm performs best in most cases with the same training set. This result occurs because—although LSHOG is better than the original HOG (as reported in [49])—the LSHOG algorithm, which computes a histogram of gradient orientations over the entire face at each pixel location, ignores the interactions between different sub-regions, causing its recognition rate to be below that of the proposed MSR-MSF-VQ algorithm. Therefore, we can conclude that the recognition performance of our proposed algorithm is more robust than that of other methods.

### Experiments on the Yale-B face database

Some additional experimental results on the Yale-B face database are provided in this section. In contrast to the Yale face database, the Yale-B face database [34, 35, 52] was constructed to test the performance of facial recognition algorithms under larger variations in lighting and pose; hence, the Yale-B face database is even more challenging. In total, it contains images of 38 individuals in 9 poses with 64 different illuminations per pose (The facial images in the Yale-B face database are copyrighted, which limits the publication of these facial images in PLOS ONE for commercial use. Consequently, in this paper, we have removed the image samples from the Yale-B face database). For our experiments, we selected only the frontal-face images of ten individuals under 64 different illumination conditions. All the images were greyscale and normalized to 168×192 pixels. We divided the 640 frontal images into five subsets based on the angle between the light source direction and the camera axis: Subset 1 (angle < 12 degrees), Subset 2 (13 < angle < 25 degrees), Subset 3 (26 < angle < 50 degrees), Subset 4 (51 < angle < 77 degrees), Subset 5 (angle > 78 degrees). The sizes of these five subsets are 70, 120, 120, 140 and 190, respectively. We conducted the two experiments to evaluate the validity of our proposed algorithm. Tables 12 and 13 show performance comparisons of the MSR-MSF-VQ algorithm and other comparable algorithms in terms of recognition performance for Experiment 1 and Experiment 2, respectively.

**Table 12:** Performance comparison of the first experiment (results of our proposed algorithm are in bold).

Approach	Subset 2 (%)	Subset 3 (%)	Subset 4 (%)	Subset 5 (%)
Raw image	95.83	76.67	46.67	24.24
HEQ	100	97.5	75	60
wavelet-based normalization	100	100	94.76	90.83
MSR-MSF-VQ	100	97.5	66.4	21.6
**MSR-MSF-VQ (SVM)**	**100**	**98.3**	**68.6**	**75.3**

**Table 13:** Performance comparison of the second experiment (results of our proposed algorithm are in bold).

Approach	Subset 1 (%)	Subset 2 (%)	Subset 3 (%)	Subset 4 (%)	Subset 5 (%)
ORI	98.6	93.3	43.3	17.9	10.5
HE	100	99.2	73.3	42.1	43.2
RG	100	100	94.2	59.3	39.5
LTV	100	100	75.8	72.1	79.8
GradFace	100	100	99.2	94.3	98.9
MSR-MSF-VQ	100	100	83.3	54.3	17.4
**MSR-MSF-VQ (SVM)**	**100**	**100**	**86.7**	**58.6**	**65.3**

1. In our first experiment, we select Subset 1, including 7 images for each person as the gallery set (the images of Subset 1 were acquired under good illumination conditions) and randomly choosing facial images from the remaining four subsets as the probe set, which is the same approach as [53]. We compare our proposed algorithm with three existing methods: Raw image (the image without any preprocessing), HEQ (histogram-equalized method) and wavelet-based normalization [53].
2. In our second experiment, the images with the most neutral light condition (“A+00E+00”) are used as the gallery set, and images from Subsets 1–5 are randomly chosen as the probe set, as in [54]. We compare our proposed MSR-MSF-VQ algorithm with several state-of-the-art methods: HE [55], LTV [56], Gradientface (GradFace) [57] and RG [58]. In addition, the result on the original image without any preprocessing (ORI) are also presented.

From the experimental results listed in Table 12 and Table 13, we can see that our proposed MSR-MSF-VQ algorithm achieved excellent recognition performance on the images with slight or moderate light variations. This result occurs because the test images that were taken under relatively good illumination conditions are more similar to the images in the gallery set. However, the worst facial recognition results occurred on the images with severe light variations. Here, our algorithm was only weakly competitive with some of the other methods on the same probe set because the images were not preprocessed to normalize the illumination effects. Therefore, it is difficult to extract an appropriate illumination-invariant feature from the images in the test set with intense shadows that were taken under poor illumination conditions. We took this factor into account and utilized the histogram equalization method to preprocess the face images of the probe sets with severe light variations (Subset 4 and Subset 5). The corresponding experimental results obtained after preprocessing using the SVM classifier are shown in Table 12 and Table 13. Preprocessing improved the recognition rate of our algorithm, especially for Subset 5, for which the recognition rate improved significantly compared with the original results. However, the improvement in the recognition results did not exceed the improvement observed when using all of the compared methods. From these results, we can still conclude that although the MSR-MSF-VQ algorithm is not robust to severe illumination variations, it achieves an excellent recognition rate under varied illumination conditions in small-scale face databases. To some extent, these results further validate the effectiveness of our proposed MSR-MSF-VQ algorithm.

In summary, the substantial comparative analysis of our proposed MSR-MSF-VQ algorithms with several state-of-the-art methods on three standard face databases performed in this work, clearly reflect the feasibility and effectiveness of the MSR-MSF-VQ algorithm under challenges of varying facial expressions, pose and illuminations. We can attribute our algorithm's satisfactory recognition performance to the extended vector quantization histogram features (the MSR-MSF-VQ feature), which not only contain the spatial structural information but also consider the significance of location information and the interactions between different facial sub-regions.

### Experiments on the CAL-PEAL-R1 face database

Finally, we conducted additional experiments on the well-known large-scale CAS-PEAL-R1 face database [36, 37]. This database contains 30,900 images of 1,040 subjects with varying accessories, expressions and lighting. The standard evaluation protocol uses all the frontal-face images. The gallery set (GS) consists of 1,040 face images: one image per subject under standard conditions. We chose three representative probe sets (images with accessories, varied expressions, and varied lighting) for our experiments. The expression set (PE) contains 1,570 face images of 377 subjects; the accessory set (PA) contains 2,285 face images of 438 subjects; and the lighting set (PL) contains 2,243 face images of 233 subjects. All the face images in the gallery and probe sets were aligned and cropped to 64×64 pixels based on the eye coordinates given in the current release of the CAS-PEAL-R1 face database (The facial images in the CAS-PEAL-R1 face database are copyrighted, which limits the publication of these facial images in PLOS ONE for commercial use. Consequently, in this paper, we have removed the image samples from the CAS-PEAL-R1 face database).

The optimal accuracy recognition rate of different approaches and our proposed algorithm for the three probe sets from CAS-PEAL-R1 face database as described above are listed in Table 14. The compared approaches are as follows:

1. Total Variation based Quotient Image model (TVQI) [59]. This is an effective method for face recognition under low-level lighting conditions.
2. The TV_L1 and TV_L2 models [59] in INM [60] (INM uses the anisotropic diffused TV_L1 model to decompose the face sample into a low-frequency part and a high-frequency part, and it uses the TV_L2 model to generate a noiseless large-scale part). The symbols “TV_L1+HE” and “TV_L2+HE” stand for the INM method implemented using the TV_L1 and TV_L2 models with histogram equalization, respectively, while “TV_L2+RHE” represents the INM implemented using the TV_L2 model with region-based histogram equalization.

**Table 14:** The maximal accuracy recognition rates (%) on the CAS-PEAL-R1 face database.

Approach	Accessory	Expression	Lighting
TVQI	43.41	60.06	10.97
TV_L+HE	46.48	61.27	7.40
TV_L+HE	47.27	61.02	7.31
TV_L+RHE	48.23	58.34	8.69
MSR-MSF-VQ-Manhattan	66.6	90.8	6.7
**MSR-MSF-VQ-SVM**	**70.68**	**93.69**	**11.41**

As shown in Table 14, our MSR-MSF-VQ algorithm clearly achieves the highest recognition rate on the accessory and expression probe sets, but it lags the other methods under low-level lighting conditions. Our MSR-MSF-VQ algorithm is obviously not beneficial on the lighting probe set. This result occurs because the TVQI model uses the low-frequency parts of the image to normalize the illumination effect in the face sample and then generates an illumination-invariant small-scale image. Furthermore, INM is an image preprocessing method that can be used to remove illumination effects in face samples, including diffuse reflections, specular reflections, cast shadows and attached shadows. By using the INM based methods, multi-scaled information containing adequate enhanced facial features can be extracted, and these are illumination invariant. None of the images used in this experiment were preprocessed to normalize the illumination effect, hence, the features described above for recognition under varied lighting conditions achieved better recognition performances than those of our algorithm. Consequently, we took the illumination effects into account and utilized the histogram equalization method to preprocess the face images of the lighting probe set. We also adopted the SVM classifier to optimize the recognition performance. The corresponding experimental results after preprocessing are shown in Table 14, from which we can observe that the preprocessing substantially improved the recognition rate of our algorithm; its results exceeded those of all comparable methods. The reasons our algorithm achieves a satisfactory recognition performance on the other two probe sets (accessory and expression)—beyond its innate advantages and the optimization function of the SVM classifier—involve the fact that the TVQI model is suitable for face recognition only under varied lighting conditions in small-scale face databases; the image information generated by TVQI is limited. Therefore, when it is used on a large-scale face database, it cannot discriminate between all the face samples. Moreover, the fact that the INM based methods preserve numerous facial features, for example, the wrinkles in a face sample, may decrease their recognition performances in the expression probe set. Overall, the experimental results on the CAS-PEAL-R1 face database further confirm the MSR-MSF-VQ algorithm's robustness for face recognition.

## Conclusions

In this paper, an improved face recognition algorithm called MSR-MSF-VQ was proposed. The main characteristic of the MSR-MSF-VQ model is that it captures spatial structural information to overcome the limitation of VQ histograms. Moreover, it also incorporates location information and the spatial interactions between facial sub-regions into the identification features, which improves the facial recognition performance. The proposed method was evaluated on five well-known face databases and comparisons were made with several state-of-the-art algorithms. Our algorithm's satisfactory recognition performances demonstrate its robustness for face recognition.

Although our proposed algorithm achieves excellent recognition rates using the extended VQ histogram features during face recognition, more work need to be done in the future. There are two directions worth exploring to further optimize our algorithm. First, the VQ histogram is utilized in this paper, and it is a reliable facial feature representation for face recognition; however, other histogram-based features such as LBP [61], HOG [10], and so on could be combined with MSF instead of VQ histogram features. Therefore, we plan to explore the relative merits of these approaches in future research. Second, we plan to explore additional common classification algorithms such as Nearest Distance Classifiers [19] and Neural Networks [21, 22] for face recognition to improve the performance of the proposed algorithm.

## References

[1] Turk M, Pentland A. Eigenfaces for recognition. J. Cognitive Neuroscience. 1991; 3(1): 71–86. 10.1162/jocn.1991.3.1.71 23964806

[2] Belhumeur PN, Hespanha JP, Kriegman DJ. Eigenfaces vs. fisherfaces: Recognition using class specific linear projection. IEEE Transactions on Pattern Analysis & Machine Intelligence. 1997; 19(7): 711–720.

[3] Kim HC, Kim D, Bang SY. Face recognition using LDA mixture model. Pattern Recognition Letter. 2002; 24(15): 2815–2821.

[4] Vinay A, Shekhar VS, Kumar CA, Natarajan S. Affine-scale invariant feature transform and two-dimensional principal component analysis: a novel framework for affine and scale invariant face recognition. Iet Computer Vision. 2016; 10(1): 43–59.

[5] Xiong H, Swamy MNS, Ahmad MO. Two-dimensional FLD for face recognition. Pattern Recognition. 2005; 38(7):1121–1124.

[6] Lee DD, Seung HS. Algorithms for non-negative matrix factorization. Adv. Neural Inform. Process. Systems. 2000; 32(6): 556–562.

[7] He XF, Niyogi P. Locality preserving projections. Proceedings of the Neural Information Processing Systems. 2004: 1059–1071.

[8] Wang J, Ma Z, Zhang B, Qi M, Kong J. A structure-preserved local matching approach for face recognition. Pattern Recognition Letters. 2011; 32(3): 494–504.

[9] Ahonen T, Hadid A, Pietikäinen M. Face Recognition with Local Binary Patterns. Springer Berlin Heidelberg. 2004; 3021: 469–481.

[10] Déniz O, Bueno G, Salido J, Torre FDL. Face recognition using Histogram of Oriented Gradients. Pattern Recognition Letters. 2011; 32 (12): 1598–1603.

[11] Albiol A, Monzo D, Martin A, Sastre J, Albiol A. Face recognition using HOG-EBGM. Pattern Recognition Letters. 2008; 29(10): 1537–1543.

[12] Wiskott L, Fellous JM, Krüger N, Malsburg CVD. Face recognition by elastic bunch graph matching. IEEE Transactions on Pattern Analysis & Machine Intelligence. 1997; 19(7): 775–779.

[13] Križaj J, Štruc V, Pavešić N. Adaptation of SIFT Features for Robust Face Recognition. Int'l Conf. on Image Analysis and Recognition (ICIAR 2010). 2010; 6111(2): 394–404.

[14] Hafed ZM, Levine MD.Face Recognition Using the Discrete Cosine Transform. International Journal of Computer Vision. 2001; 43(3):167–188.

[15] Wen Y. An improved discriminative common vectors and support vector machine based face recognition approach. 2012; 39(4): 4628–4632.

[16] Wright J, Yang AY, Ganesh A, Sastry SS, Ma Y. Robust face recognition via sparse representation. IEEE Trans Pattern Anal Mach Intell. 2009; 31(2): 210–27. doi: 10.1109/TPAMI.2008.79 19110489 10.1109/TPAMI.2008.79

[17] Naseem I, Togneri R, Bennamoun M. Linear Regression for Face Recognition. IEEE Trans. PatternAnal. Mach. Intell. 2010; 32(11): 2106–2112. 10.1109/TPAMI.2010.128 20603520

[18] Hu C, Ye M, Du Y, Lu X. Vector projection for face recognition. Computers & Electrical Engineering. 2014; 40(8):51–65.

[19] Shen F, Hasegawa O. A fast nearest neighbor classifier based on self-organizing incremental neural network. Neural Networks. 2008; 21(10): 1537–1547. doi: 10.1016/j.neunet.2008.07.001 18678468 10.1016/j.neunet.2008.07.001

[20] Moghaddam B, Nastar C, Pentland A. A bayesian similarity measure for direct image matching. Proc. Internat. Conf. on Pattern Recognition. 1996; 2(5): 350–358.

[21] Wen Y, Zhang K, Li Z, Qiao Y. A Discriminative Feature Learning Approach for Deep Face Recognition. European Conference on Computer Vision (ECCV 2016). 2016:499–515.

[22] Krizhevsky A, Sutskever I, Hinton GE. ImageNet classification with deep convolutional neural networks. International Conference on Neural Information Processing Systems. 2012; 25(2):1097–1105.

[23] Berg A, Deng J, Fei-Fei L (2010) Large scale visual recognition challenge 2010. www.imagenet.org/challenges.2010

[24] Martez AM, Kak AC. PCA versus LDA. IEEE Trans. Pattern Anal. Machine Intell. 2001; 23(2): 228–233.

[25] Kotani K, Chen Q, Ohmi T. Face Recognition Using Vector Quantization Histogram Method. Int'l Conf. on Image Processing. 2002; 2: 105–108.

[26] “The ORL Database of Faces,” at http://www.cl.cam.ac.uk/research/dtg/attarchive/facedatabase.html

[27] Li J, Wu W, Wang T, Zhang Y. One step beyond histograms: Image representation using Markov Stationary features. Proc. of the IEEE Conference on Computer Vision and Pattern Recognition (CVPR’08).2008: 1–8.

[28] Yan Y, Chen Q, Lee FF. Face Recognition Using Extended Vector Quantization Histogram Features. Int'l Conf. on Signal and Image Processing (ICSIP 2016). 2016: 90–95.

[29] Samaria FS, Harter AC. Parameterisation of a stochastic model for human face identification. IEEE Workshop on Applications of Computer Vision. 1994; 22: 138–142.

[30] Phillips PJ, Moon H, Rizvi SA, Rauss PJ. The FERET evaluation methodology for face-recognition algorithms. IEEE Transactions on Pattern Analysis and Machine Intelligence 2000; 22(10): 1090–1104.

[31] Phillips PJ, Wechsler H, Huang J, Rauss PJ. The FERET database and evaluation procedure for face-recognition algorithms. Image and Vision Computing. 1998; 16(5): 295–306.

[32] Martinez M and Benavente R. The AR Face Database. CVC Technical Report. 1998.

[33] “Yale face database,” at http://vision.ucsd.edu/content/yale-face-database

[34] Lee KC, Ho J, Kriegman DJ. Acquiring Linear Subspaces for Face Recognition under Variable Lighting. IEEE Trans. Pattern Anal. Mach. Intelligence. 2005; 27(5): 684–698. 10.1109/TPAMI.2005.92 15875791

[35] Georghiades AS, Belhumeur PN, Kriegman DJ. From Few to Many: illumination cone models for face recognition under variable lighting and pose. IEEE Trans. Pattern Anal. Mach. Intelligence. 2001; 23(6): 643–660.

[36] “CAS-PEAL Face Database,” at http://www.jdl.ac.cn/peal/index.html

[37] Gao W, Cao B, Shan S, Chen X, Zhou D, Zhang X, et al The CAS-PEAL Large-Scale Chinese Face Database and Baseline Evaluations. IEEE Trans. on System Man, and Cybernetics (Part A). 2008; 38(1): 149–161.

[38] FERET face database, https://www.nist.gov/programs-projects/face-recognition-technology-feret

[39] Yan Y, Lee FF, Chen Q. Improved Face Recognition Algorithm Using Extended Vector Quantization Histogram Features. Int'l Conf. on Signal Processing (ICSP 2016). 2016: 1046–1050. 10.1371/journal.pone.0190378 PMC5749794 29293581

[40] Fan RE, Chang KW, Hsieh CJ, Wang XR, Lin CJ. LIBLINEAR: A Library for Large Linear Classification. Journal of Machine Learning Research. 2012; 9(9):1871–1874.

[41] AR face database, http://www2.ece.ohio-state.edu/~aleix/ARdatabase.html

[42] Gottumukkal R, Asari VK. An improved face recognition technique based on modular PCA approach. Pattern Recognition Letter. 2004; 25(4): 429–436.

[43] Chen S, Zhu Y. Subpattern-based principle component analysis. Pattern Recognition. 2004; 37(5): 1081–1083.

[44] Tan K, Chen S. Adaptively weighted sub-pattern PCA for face recognition. 2005; 64(1): 505–511.

[45] Kumar KV, Negi A. SubXPCA and a generalized feature partitioning approach to principal component analysis. Pattern Recognition. 2008; 41(4): 1398–1409.

[46] Zhu YL. Sub-pattern non-negative matrix factorization based on random subspace for face recognition. 2007; 3: 1356–1360.

[47] Cai D, He X, Hu Y, Han J. Learning a spatially smooth subspace for face recognition. IEEE Conf. on Computer Vision and Pattern Recognition. 2007: 1–7.

[48] Wang J, Zhang B, Wang S, Qi M, Kong J. An adaptively weighted sub-pattern locality preserving projection for face recognition 2010; 33(3): 323–332.

[49] Li B, Huo G. Face recognition using locality sensitive histograms of oriented gradients. International Journal for Light and Electron Optics. 2015; 127(6): 3489–3494.

[50] Yan S, Xu D, Zhang B, Zhang HJ, Yang Q, Lin S. Graph Embedding And Extension: A General Framework For Dimensionality Reduction. IEEE Trans. Pattern Anal. Mach. Intell. 2007; 29 (1): 40–51. doi: 10.1109/TPAMI.2007.12 17108382 10.1109/TPAMI.2007.12

[51] He X, Cai D, Yan S, Zhang HJ. Neighborhood Preserving Embedding. Proceedings of the 10th IEEE International Conference on Computer Vision. 2005; 2(23): 1208–1213.

[52] YALE-B face database, http://vision.ucsd.edu/~iskwak/ExtYaleDatabase/ExtYaleB.html

[53] Du S, Ward R. Wavelet-based illumination normalization for face recognition. Proc. of IEEE Int'l Conf. on Image Processing (ICIP 2005). 2005; 2: 954–957.

[54] Wang B, Li W, Yang W, Liao Q. Illumination Normalization Based on Weber's Law with Application to Face Recognition. IEEE Signal Processing Letters. 2011; 18(8): 462–465.

[55] Pizer SM, Amburn EP, Austin JD, Cromartie R, Geselowitz A, Greer T et al Adaptive histogram equalization and its variations. Comput. Vis. Graph. Image Process. 1987; 39(3): 355–368.

[56] Chen T, Yin W, Zhou XS, Comaniciu D, Huang TS. Total variation models for variable lighting face recognition. IEEE Trans. PatternAnal. Mach. Intell. 2006; 28(9): 1519–1524. 10.1109/TPAMI.2006.195 16929737

[57] Zhang T, Tang YY, Fang B, Shang Z, Liu X. Face recognition under varying illumination using gradientfaces. IEEE Trans. Image Process. 2009); 18(11): 2599–2606. doi: 10.1109/TIP.2009.2028255 19635700 10.1109/TIP.2009.2028255

[58] Hou Z, Yau WY. Relative gradients for image lighting correction. ICASSP. 2010: 549–556.

[59] Chen T, Yin W, Zhou XS, Comaniciu D, Huang TS. Illumination Normalization for Face Recognition and Uneven Background Correction Using Total Variation Based Image Models. Proc. IEEE Internat. Conf. on Computer Vision and Pattern Recognition. 2005; 2(2): 532–539.

[60] An G, Wu J, Ruan Q. An illumination normalization model for face recognition under varied lighting conditions. Pattern recognition Letters. 2010; 31(9):1056–1067.

[61] Ojala T, Pietikäinen M, and Harwood D. A Comparative Study of Texture Measures with Classification Based on Feature Distributions. Pattern Recognition. 1996; 29: 51–59.

